# Endoscopic retrograde direct cholangioscopy combined with endoscopic papillectomy in treatment of duodenal papillary adenoma

**DOI:** 10.1055/a-2665-7633

**Published:** 2025-08-27

**Authors:** Shan-Shan Hu, Hong Shen, Yun-Chao Yang, Jie Hou, Wei-Hui Liu

**Affiliations:** 1 Department of Gastroenterology and Hepatology, Sichuan Provincial People’s Hospital, School of Medicine, University of Electronic Science and Technology of China, Chengdu, China

Given the high risk of malignant transformation in duodenal papillary adenomas, timely endoscopic resection is critical 1 2 . The conventional procedure involves snare resection followed by placement of biliary and pancreatic duct stents under fluoroscopic guidance to ensure drainage. However, conventional radiographic methods cannot assess invasion at the terminal ends of the biliary and pancreatic ducts, and in some cases, cannulation of these ducts may fail. To address these issues during endoscopic procedures, we apply endoscopic retrograde direct cholangioscopy (ERDC) 3 4 , a technique that we developed to allow complete visual resection of duodenal papillary adenomas without fluoroscopic assistance.

A female patient presented with a duodenal papillary adenoma. Complete resection was successfully achieved using snare polypectomy ( Fig. 1 ), with prophylactic application of hemostatic clips to the resection site to minimize the risk of delayed bleeding ( Fig. 2 ). We then employed a conical transparent cap-fitted cholangioscope to enable direct visualization during biliary and pancreatic duct cannulation. Subsequently, the distal ends of the common bile duct and pancreatic duct were examined under direct cholangioscopic vision to exclude residual tumor ( Fig. 3 ), followed by placement of plastic stents ( Fig. 4 ). Histopathological analysis confirmed a tubulovillous adenoma with clear basal and lateral margins ( Fig. 5 ). The patient recovered well without complications and was discharged uneventfully ( Video 1 ).

**Fig. 1:**
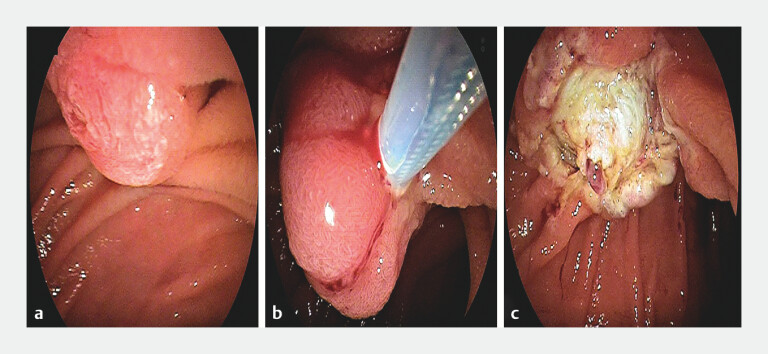
Endoscopic resection of a duodenal papillary adenoma. **a** Duodenal papillary adenoma. **b** Snare resection of the lesion. **c** Postoperative wound.

**Fig. 2:**
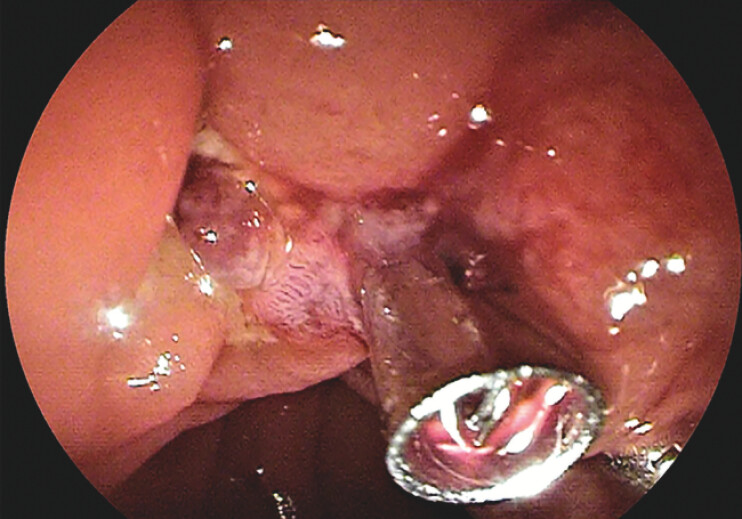
Endoscopic clip closure of the distal part of the surgical wound.

**Fig. 3:**
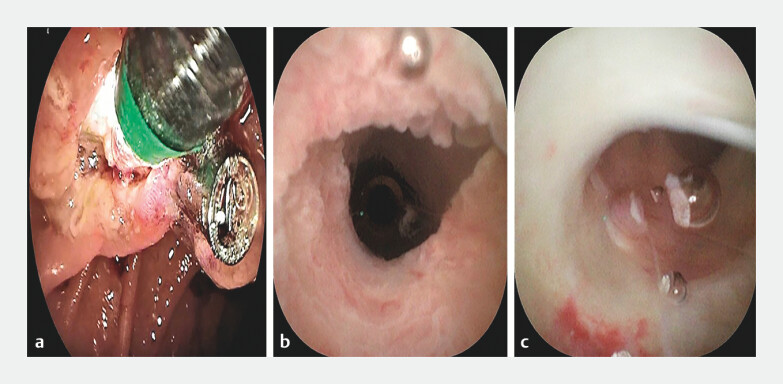
Endoscopic retrograde direct cholangioscopy (ERDC) technology for direct visual inspection of biliary and pancreatic ducts. **a** A conical transparent cap was installed at the distal end of the cholangioscope. **b** ERDC confirmed that there was no residual tumor in the bile duct. **c** There was no residual tumor in the pancreatic duct orifice under direct vision.

**Fig. 4:**
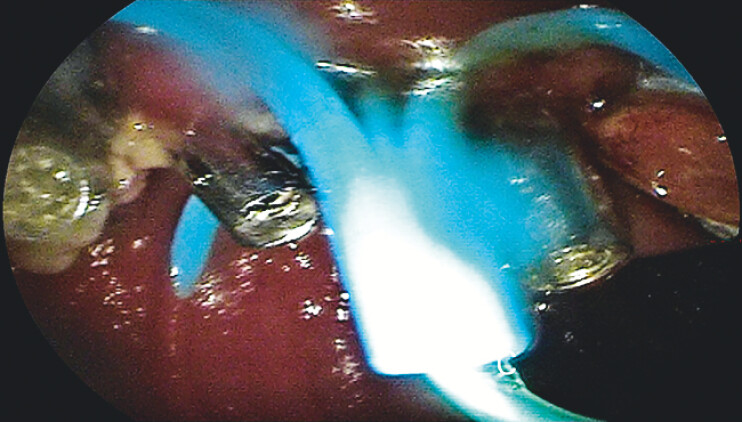
Indwelling biliary and pancreatic duct stents.

**Fig. 5:**
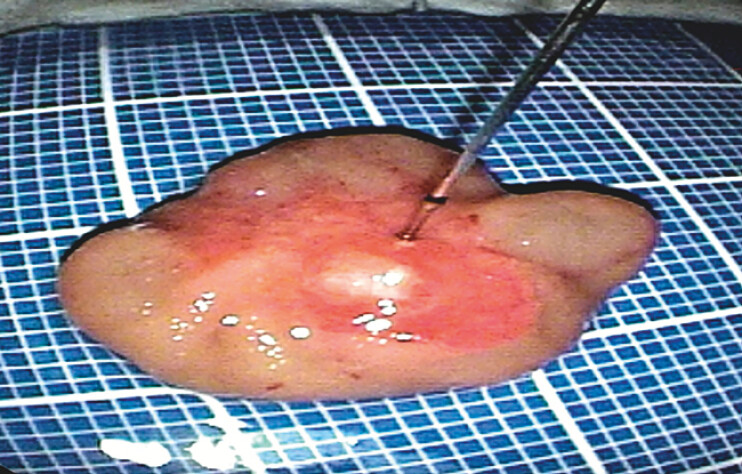
Postoperative specimen was fixed on a foam panel.

The endoscopic retrograde direct cholangioscopy technique can not only determine whether there is residual tumor in the biliary and pancreatic ducts but also ensure the effectiveness of biliary and pancreatic stent placement.Video 1

This case demonstrates that in specific patient populations, such as those with duodenal papillary adenoma, including pregnant women and children, the ERDC technique can be utilized to achieve complete radiation-free endoscopic treatment, thereby avoiding radiation exposure. The radiation-free papillary resection procedure is safe and effective. The ERDC technique can not only determine whether there is residual tumor in the biliary and pancreatic ducts but also ensure the effectiveness of biliary and pancreatic stent placement.

Endoscopy_UCTN_Code_TTT_1AR_2AF

## Notes

Endoscopy E-Videos https://eref.thieme.de/e-videos
*E-Videos* is an open access online section of the journal *Endoscopy* , reporting on interesting cases and new techniques in gastroenterological endoscopy. All papers include a high-quality video and are published with a Creative Commons CC-BY license. Endoscopy E-Videos qualify for HINARI discounts and waivers and eligibility is automatically checked during the submission process. We grant 100% waivers to articles whose corresponding authors are based in Group A countries and 50% waivers to those who are based in Group B countries as classified by Research4Life (see: https://www.research4life.org/access/eligibility/ ). This section has its own submission website at https://mc.manuscriptcentral.com/e-videos .

## References

[1] Hollenbach M Heise C Abou-Ali E Endoscopic papillectomy versus surgical ampullectomy for adenomas and early cancers of the papilla: a retrospective Pancreas2000/European Pancreatic Club analysis Gut 2025 74 397 409 10.1136/gutjnl-2022-327996 39642968

[2] Lv Y Wang P Chen J Indicative value of pathological classification of duodenal papillary adenomas in clinical diagnosis and treatment Surg Endosc 2022 36 5183 5197 10.1007/s00464-021-08894-0 35286472

[3] Liu WH Huang XY Hu X Initial experience of visualized biliary cannulation during ERCP Endoscopy 2023 55 1037 1042 37339664 10.1055/a-2113-8952

[4] Liu WH Huang XY Zhang RY From darkness to brightness: the cholangioscopy-guided selective biliary cannulation with the help of transparent cap during ERCP Endoscopy 2023 55 E320 E321 36513111 10.1055/a-1981-2503 PMC9833945

